# A proteomic insight into the midgut proteome of *Ornithodoros moubata* females reveals novel information on blood digestion in argasid ticks

**DOI:** 10.1186/s13071-017-2300-8

**Published:** 2017-08-01

**Authors:** Ana Oleaga, Prosper Obolo-Mvoulouga, Raúl Manzano-Román, Ricardo Pérez-Sánchez

**Affiliations:** Parasitology Laboratory, Institute of Natural Resources and Agrobiology (IRNASA, CSIC), Cordel de Merinas, 40-52, 37008 Salamanca, Spain

**Keywords:** *Ornithodoros moubata*, Soft tick, Midgut, Proteome, Blood digestion

## Abstract

**Background:**

The argasid tick *Ornithodoros moubata* is the main African vector of the human relapsing fever agent *Borrelia duttoni* and the African swine fever virus. Together with saliva, the tick midgut forms part of the host-tick-pathogen interface, and numerous midgut proteins play key functions in the blood digestion-related process and the infection and transmission of pathogens. This work explores the composition of the midgut proteome of unfed and fed *O. moubata* females with the aim to complete the biological information already obtained from the midgut transcriptome and provide a more robust and comprehensive perspective of this biological system.

**Methods:**

Midgut tissues taken from females before feeding and 48 h after feeding were subjected to LC/MS-MS analysis. After functional characterization and classification of the proteins identified, the differences in the proteome between unfed and fed females were analysed and discussed. Additionally, a detailed analysis of particular groups of proteins that are involved in the processes of nutrient digestion and responses to the oxidative stress was carried out.

**Results:**

1491 non-redundant tick proteins were identified: 1132 of them in the midgut of unfed ticks, 1138 in the midgut of fed ticks, and up to 779 shared by both physiological conditions. Overall, the comparative analysis of the midgut proteomes of *O. moubata* females before and after feeding did not reveal great differences in the number or class of proteins expressed, enzymatic composition or functional classification.

**Conclusions:**

The hemoglobinolytic system in ixodids and argasids is very similar in spite of the fact that they display very different feeding and reproductive strategies. Although the main source of nutrients in ticks are proteins, lipids and carbohydrates also constitute significant nutritional sources and play an important part in the process of blood digestion. The genes and proteins involved in intracellular transport mechanisms, defensive responses, detoxifying responses and stress responses seem to be closely regulated, highlighting the complexity and importance of these processes in tick biology, which in turn assigns them a great interest as targets for therapeutic and immunological interventions.

**Electronic supplementary material:**

The online version of this article (doi:10.1186/s13071-017-2300-8) contains supplementary material, which is available to authorized users.

## Background

Ticks are haematophagous ectoparasites that classify into two main families, the Ixodidae (hard ticks) and the Argasidae (soft ticks). Both families differentiate in a range of morphological and biological characteristics, for example, their feeding and reproductive strategies. Typically, ixodids are slow feeders that take several days to ingest their blood meal, while argasids are fast feeders that complete their blood meal in minutes to hours. Ixodid females feed only once, lay thousands of eggs and die after oviposition, whereas adult argasids can feed and reproduce up to ten times, laying hundreds of eggs per trophogonic cycle [1–5].

Both tick families represent important threats for human and animal health because they cause direct damage to hosts (including paralysis, toxicosis, irritation and allergy) and transmit a wide range of pathogenic microorganisms to them [4, 6]. This noteworthy vector capacity together with tick abundance has placed ticks as the most important arthropod vectors of diseases affecting livestock, pets and people [7].

The argasid *Ornithodoros moubata* is distributed throughout southern and eastern African countries [8], where it transmits the African swine fever virus and the East African human relapsing fever causing agent, *Borrelia duttoni* [9, 10].

Chemical acaricide-based tick control has shown important drawbacks, including a selection of tick resistant strains, environmental pollution and animal product contamination, which have encouraged the search for alternative methods of tick control, such as immunological control with anti-tick vaccines [11].

With this purpose some years ago, our team started to develop an anti-*O. moubata* vaccine by testing two types of candidate antigens: salivary antigens, which are naturally exposed to the host immune system during tick feeding; and concealed antigens from the intestinal tissue. The studies on salivary antigens identified three protective anti-haemostatic molecules, which when jointly administered to rabbits provided more than 50% vaccine efficacy, making them promising vaccine candidates. Despite this, a fully protective vaccine to *O. moubata,* based on salivary antigens only, has not been hitherto obtained [12].

On the other hand, the possibility of inducing protective immune responses to intestinal (midgut) antigens of *O. moubata* seemed reasonably high, since such antigens (namely, the Bm86 glycoprotein from *Rhipicephalus microplus*) were the basis of the two anti-tick vaccines marketed to date (TickGARD® and GAVAC®) [13].

Together with tick saliva, the tick midgut forms part of the host-tick-pathogen interface, and numerous midgut proteins play key functions in the blood digestion-related process and the infection and transmission of pathogens. Thus, midgut proteins are considered potential targets for the development of new drugs and vaccines aimed at tick control [14–16].

In this direction, our former studies with *O. moubata*, and with the Iberian species *O. erraticus*, demonstrated that vaccination of animals with extracts of midgut membrane proteins induced protective responses. These responses significantly reduced female feeding and reproduction rates in both species, and the survival rates in *O. erraticus* nymphs, confirming the potential of the midgut as a source of protective antigens [17, 18].

In ixodid ticks, the midgut molecular machinery for blood digestion, as well as the proteins involved in successful pathogen transmission, have been reasonably well established [19–25]. In argasids, by contrast, this topic has been little explored, and the information available is scant. Only the identification of several proteolytic enzymes and some proteins associated with defence responses, such as lysozyme and several defensins, have been reported in the midgut of *O. moubata* [26, 27]. This paucity of information on the argasid midgut, and the potential of proteins involved in physiological processes that take place in the tick midgut as vaccine antigens prompted us to the study the molecular machinery involved in blood digestion in argasids. With this aim, our first step was the characterization of the midgut proteome of *O. erraticus* females before and after the blood meal. This study described the first midgut proteome in argasids and provided interesting novel information on blood ingestion-induced changes in the expression of midgut proteins in this species, contributing to the understanding of the multi-enzymatic molecular system for blood digestion in argasids [28]. More recently, the midgut transcriptomes of *O. moubata* females before and after the blood meal have been assembled and analysed, and the genes that were differentially expressed in the midgut after feeding have been identified and functionally annotated. This study has significantly expanded our knowledge of the biochemistry and physiology of blood digestion in argasids [29].

In the current work, our purpose was to investigate the composition of the midgut proteomes of *O. moubata* females before and after blood meal ingestion. This will complete the biological information already obtained from the midgut transcriptome and provide a more robust and comprehensive perspective of this biological system. To reach this objective, we have applied an experimental approach similar to that previously used by Oleaga et al. [28] for *O. erraticus.* Thus, midgut extracts from fed and unfed females were obtained and fractionated into soluble and membrane proteins; SDS-PAGE then resolved the fractions, and the gels were sliced into pieces. The proteins in each gel slide were subject to LC-MS/MS followed by searching the NCBInr_metazoa and EST_acari databases for protein identification. Additionally, to maximize the number of protein identifications, a proteomics informed by transcriptome (PIT) analysis was also implemented, which consisted of mining the *O. moubata* midgut transcriptomic dataset previously obtained by RNAseq [29] as an additional database.

## Methods

### Ticks and collection of tick midgut samples

The *O. moubata* specimens were obtained from the laboratory colony of the IRNASA (CSIC), which is regularly fed on rabbits and maintained at 28 °C, 85% relative humidity and a 12/12 h light/dark photoperiod.

Homogeneous batches of *O. moubata* females taken before feeding (unfed) and at 48 h post-feeding (fed) were dissected in cold phosphate buffered saline (PBS) pH 7.4. The midguts were recovered, rinsed several times with PBS to eliminate host blood and tick hemolymph proteins and inspected to remove contaminant tissues, mostly Malpighian tubules and tracheae [18].

### Preparation of midgut protein extracts from unfed and fed females

We followed the procedure previously described by Oleaga et al. [28]. Briefly, batches of 25 midguts were suspended and homogenized in ice-cold PBS with proteinase inhibitors (Roche Diagnostics, Indianapolis, USA), followed by sonication (6 pulses, 30 s/pulse) (Ultrasonic Cell Disrupter Virsonic 300). Tissue homogenates were centrifuged for 20 min at 10,000× *g* and 4 °C to remove particulate remnants, and the supernatants were re-centrifuged for 1 h at 100,000× *g*, and 4 °C. Final supernatants were recovered and named as the S fraction (soluble proteins). The pellets were re-suspended in PBS and recentrifuged for 1 h at 100,000 g and 4 °C. These final pellets, enriched in membrane proteins, were recovered and named as the M fraction (membranes). The protein concentrations in these samples were assessed using the BCA Protein Assay Reagent kit (Thermo-Fisher, Rockford, USA). Samples were stored at -20 °C.

### Sample fractioning by SDS-PAGE

The four samples of midgut proteins, namely, soluble and membrane-associated proteins from unfed (S-0, M-0) and fed (S-1, M-1) females were resolved by 5–20% gradient SDS-PAGE. Gels were loaded with 20 μg of protein sample per lane and stained with Sypro Ruby (Bio-Rad, Hercules, USA) for protein visualisation and image analysis (ChemiDoc System and Image Lab software, Bio-Rad). Replicated gels were stained with Coomassie Blue. In these gels, each lane (corresponding to a different sample) was sliced into 10 pieces, and the resulting 40 slices were each subjected to protein digestion and mass spectrometry analysis for protein identification.

### Enzymatic digestion and liquid chromatography and tandem mass spectrometry analysis (LC-MS/MS)

Trypsin digestion and LC-MS/MS analysis were carried out at the SCSIE_University of Valencia Proteomics Unit, a member of ISCIII ProteoRed Proteomics Platform, following a standardized protocol, described previously [28].

Briefly, gel slices were reduced with DTT, alkylated with iodoacetamide and digested with 20 ng/μl of trypsin (Promega, Madison, USA) overnight at 37 °C. Digestion was stopped with 10% trifluoroacetic acid (TFA) to a final concentration of 0.1%, and the supernatants were filtered through a 0.22 μm filter and dried by centrifugation in a vacuum. Pellets were re-suspended in 6 μl of 5% acetonitrile, 0.1% TFA, and 5 μl of every sample was loaded onto a trap column (Nano LC Column, 3 μ C18-CL, 350 μm × 0.5 mm, Eksigent) and desalted with 0.1% TFA at a flow rate of 3 μl/min for 5 min. The peptides were then loaded onto an analytical column (LC Column, 3 μ C18-CL, 75 μm × 25 cm, Eksigent) and equilibrated in 5% acetonitrile (ACN) and 0.1% formic acid (FA). Elution was carried out with a linear gradient of 5–40% B (B: ACN, 0.1% FA) in A (A: 0.1% FA) for 30 min at a flow rate of 300 nl/min. Eluted peptides were analysed in a nanoESI qQTOF mass spectrometer (5600TripleTOF, ABSCIEX, Ontario, Canada) in IDA mode performing 0.25-s TOF MS scans from 350 to 1250 m/z, followed by 0.05-s product ion scans from 100 to 1500 m/z on the 50 most intense 2–5 charged ions.

### Database search and protein identification

Mass spectra were processed with Mascot v.2.2 (Matrix Science, Boston, USA) and the following databases were searched: protein sequences of the National Center for Biotechnology Information (NCBI) with taxonomic restriction to Metazoa (NCBInr_metazoa: 7,616,579 sequences); nucleotide sequences (EST) of the NCBI, restricted to subclass Acari (EST_acari: 2,476,050 sequences); and midgut transcriptome of *O. moubata* (annotated with restriction to phylum Arthropoda: 6629 sequences; [29]). Searches were performed with tryptic specificity, allowing one missed cleavage and tolerance on the mass measurement of 70 ppm in MS mode and 0.6 Da for MS/MS ions. Carbamidomethylation of Cys was used as a fixed modification and oxidation of Met and deamidation of Asn and Gln as variable modifications. The significance threshold was set at a confidence ≥95% and only proteins with at least two unique significant peptides were selected and shown in the results. Database searching was individually performed for each of the 40 gel slices, and jointly for each protein sample by combining all spectra from the 10 gel slices corresponding to each sample.

After manually inspecting all the proteins identified in the three databases, redundant identifications were removed by preferentially selecting the proteins matched to the *O. moubata* midgut transcriptome or the protein hit with the highest score. Contaminants, such as keratins and porcine trypsin, were also excluded from the lists of proteins identified.

The relative quantitation of the proteins was carried out using the exponentially modified protein abundance index (emPAI), which is based on protein coverage by the peptide matches in a database search result [30].

### Functional characterization and protein classification

The functional characterization and classification of the proteins identified were carried out according to Gene Ontology hierarchy (GO), using the UniProt tools (http://www.uniprot.org) and the PANTHER Classification System (Protein Analysis Through Evolutionary Relationships; http://www.pantherdb.org) [31]. The Kyoto Encyclopedia of Genes and Genomes (KEGG) pathways analysis was executed using the Blast2GO software (https://www.blast2go.com/).

## Results and discussion

### Midgut protein extracts

**Fig. 1:**
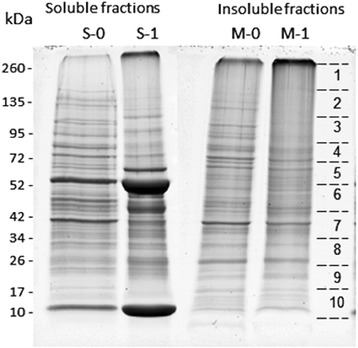
Sypro Ruby-stained 5–20% polyacrylamide gel showing the protein fractions obtained from midgut homogenates of fed and unfed *Ornithodoros moubata* ticks. Gel lanes were sliced into the ten pieces indicated at the right, and the resulting gel slices were digested with trypsin and analysed by LC-MS/MS. Lanes S-0 and S-1: supernatants from midgut homogenates from unfed and fed ticks, respectively; Lanes M-0 and M-1: pellets from midgut homogenates from unfed and fed ticks, respectively

Midgut extracts from fed and unfed *O. moubata* females were separated by centrifugation into two types of fractions: the fractions enriched in soluble proteins (supernatants S-0, S-1) and the fractions enriched in insoluble membrane-associated proteins (pellets M-0, M-1). Figure 1 shows that all four samples were complex mixtures of proteins with a broad range of molecular sizes, from 10 to 260 kDa. Band patterns were different among samples, particularly in sample S-1, which contained three very intense bands of 10, 52 and 66 kDa. This sample contained the soluble proteins from fed ticks, and these three bands corresponded to host haemoglobin, the heavy chain of immunoglobulins and serum albumin, respectively (Fig. 1).

Because of their abundance, the presence of these host proteins in the midgut samples from fed ticks could mask the identification of a significant portion of the tick proteins. This problem could also occur in unfed ticks, although to a lesser extent, since host proteins can persist for months after tick feeding and moulting [32]. This problem can be avoided by treating the sample to reduce the amount of haemoglobin, for example, by chloroform precipitation [32]. In the current work, we fractionated the samples into 10 gel slices, which were analysed individually. In this way, the abundant host blood proteins (haemoglobin, immunoglobulin and serum albumin) were concentrated in a few gel slices, avoiding the problem of these proteins hindering the detection of tick proteins present in the remaining gel slices.

### Protein identifications

The proteins shown in this section were identified by searching databases with the combined spectra of the 10 gel slices from each fraction (S-0, M-0, S-1 and M-1). To improve the number of identifications, searches were performed in three databases: two of them are publicly accessible (NCBInr_Metazoa and NCBI_EST_Acari), and the third was custom-made from the *O. moubata* midgut transcriptomic data obtained by Oleaga et al. [29].

**Fig. 2:**
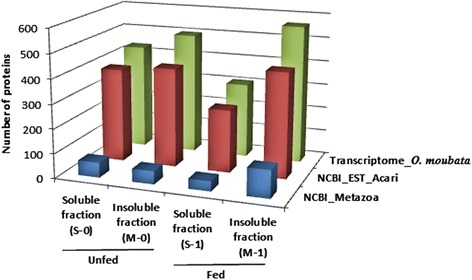
The number of proteins identified in the fractions of the midgut of unfed and fed *O. moubata* females, in each database

Table 1 and Fig. 2 summarize the number of non-redundant proteins identified in each midgut fraction in each database. Since all the samples contained host and tick proteins, the protein origin was assigned to either host or tick by applying the same criteria as Oleaga et al. [28] in their analysis of the *O. erraticus* midgut proteome. In this way, protein hits to tick and arthropod species, and to non-mammal vertebrates were all considered of tick origin, whereas protein hits to rabbit or any other mammal were considered of host origin.

**Table 1:** Number of unique proteins identified in the midgut fractions from *Ornithodoros moubata* fasted females (unfed group) and from engorged females after 48 h post-feeding (fed group). Redundant identifications and contaminants have been excluded. Soluble and insoluble fractions are the supernatants and pellets, respectively, after a centrifugation of midgut homogenates at 100,000× *g*

	Unfed ticks		Fed ticks	
	Soluble fraction (S-0)	Insoluble fraction (M-0)	Soluble fraction (S-1)	Insoluble fraction (M-1)
Tick proteins (number)				
NCBI_Metazoa	62	54	41	113
NCBI_EST_Acari	385	406	257	430
Transcriptome_*O. moubata*	433	499	307	565
Total unique tick proteins	719	813	516	908
Host proteins (number)				
NCBI_Metazoa	23	9	59	52
NCBI_EST_Acari	4	-	8	4
Total unique host proteins	26	9	67	48

Regarding proteins of host origin, the highest number of identifications arose from searching the NCBI_Metazoa database, particularly for the samples from fed ticks (59 and 52 proteins in S-1 and M-1; 23 and 9 proteins in S-0 and M-0) (Table 1). Additional file 1: Table S1 shows all the host proteins identified in every sample after searching the NCBI_Metazoa and EST_Acari databases. Since the *O. moubata* midgut transcriptome only included sequences annotated to arthropods, searching this database did not identify any protein of host origin. All host proteins were excluded from further characterization and analysis in the current work.

Regarding the proteins from *O. moubata* (Table 1, Fig. 2, Additional file 2: Table S2), the number of identifications significantly varied depending on the searched database. In the NCBI_Metazoa database, we identified between 41 and 113 proteins; in the EST-Acari database, between 257 and 430 proteins; while the highest numbers of identifications, between 307 and 565 proteins, were obtained from mining the transcriptomic sequences of the *O. moubata* midgut.

Protein identification by mass spectrometry is dependent on the available information in databases, but usually, this information is scant or negligible for non-model organisms. However, the recent development and increasing application of new generation sequencing methods (RNA-seq) provide large amounts of transcriptomic data, which once annotated can be mined for mass spectrometry-based identification of proteins and peptides. This methodological approach is known as proteomics informed by transcriptomics (PIT) analysis, which has demonstrated its utility in proteomic studies on species without a sequenced genome, such as most tick species [33–37]. Our results on *O. moubata* confirm the usefulness of PIT analysis.

**Fig. 3:**
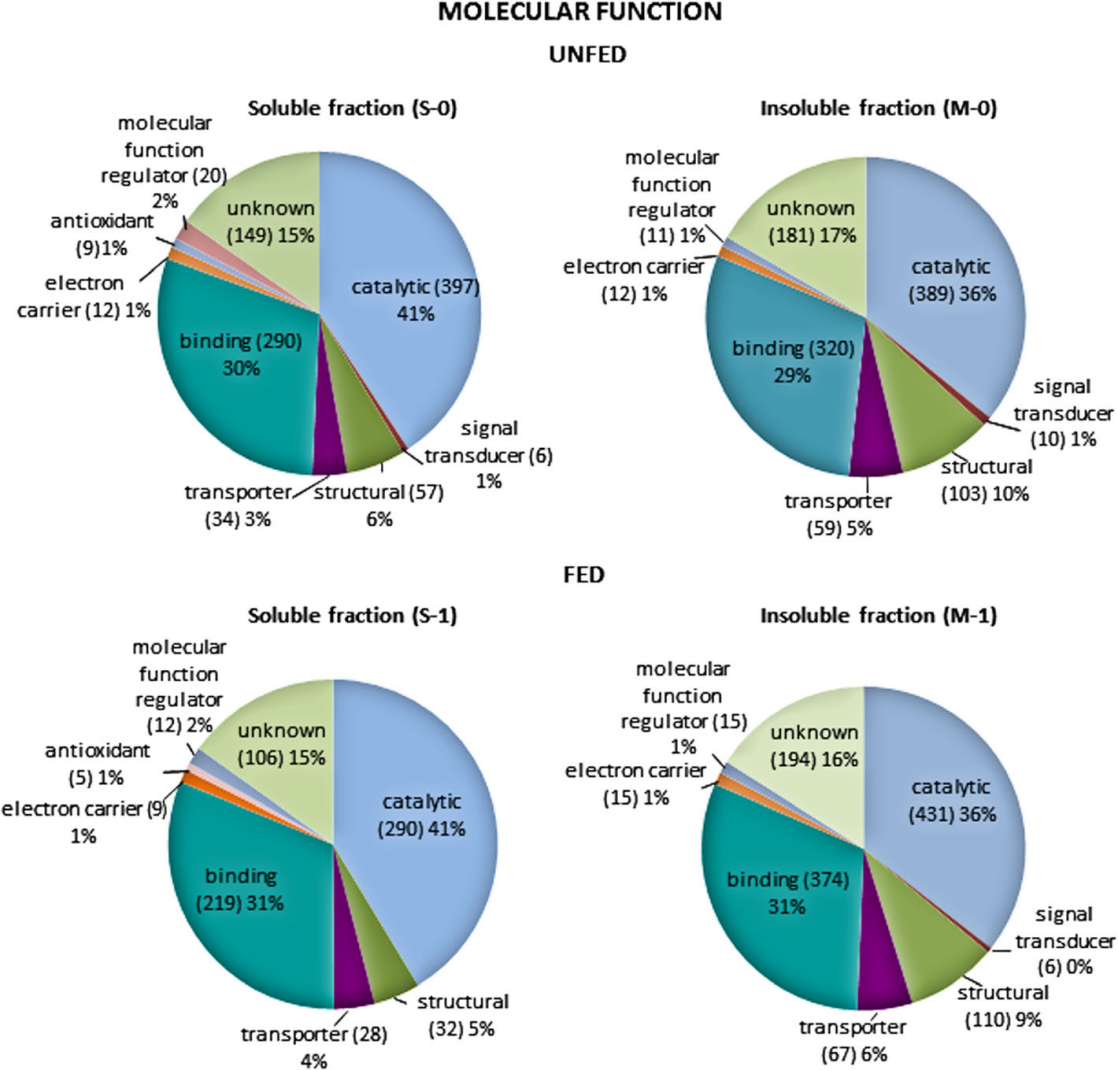
Classification according to molecular function of the proteins identified in each of the midgut fractions obtained from unfed (S-0, M-0) and fed (S-1, M-1) ticks. Only categories with more than five proteins were considered. The percentage in each category is the ratio between the number of proteins in each category, indicated in parentheses, and total proteins identified in that fraction

**Fig. 4:**
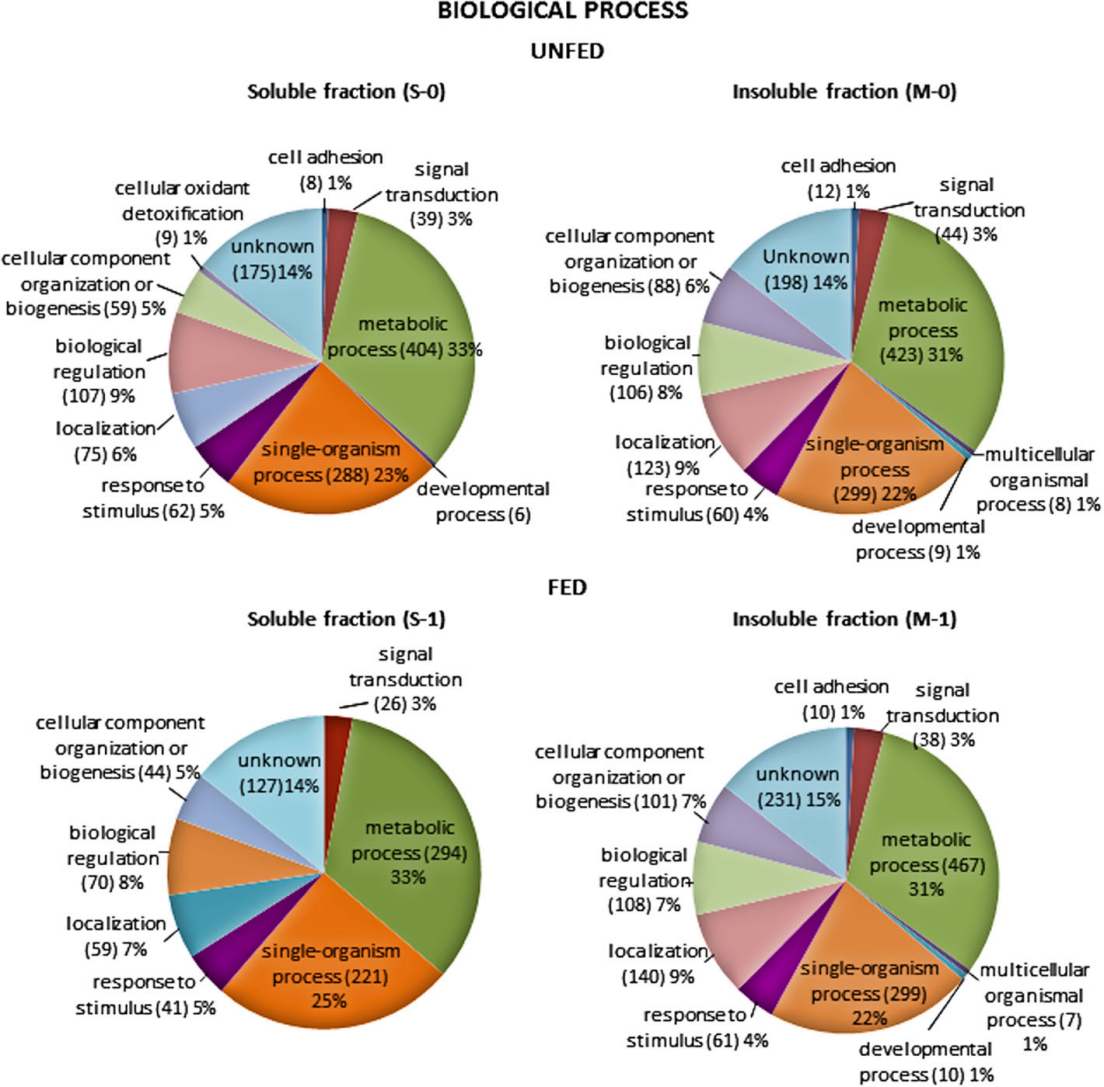
Classification according to biological process of the proteins identified in each of the midgut fractions obtained from unfed (S-0, M-0) and fed (S-1, M-1) ticks. Only categories with more than five proteins were considered. The percentage in each category is the ratio between the number of proteins in each category, indicated in parentheses, and the total proteins identified in that fraction

Whole proteins identified for each sample in the three databases were inspected, and redundant identifications were eliminated. As a result, 719 and 516 non-redundant proteins were respectively recorded for the midgut soluble fractions S-0 and S-1, while 813 and 908 non-redundant proteins were, respectively, recorded for the membrane protein-enriched insoluble fractions M-0 and M-1 (Table 1, Additional file 2: Table S2). After that, the proteins identified in each fraction were classified according to their assigned molecular function and biological process in the “Gene Ontology” database. Protein categories including less than five proteins were excluded from further analyses (Figs. 3 and 4).

Regarding the classification of molecular function, the more abundant categories in the four samples were the proteins with catalytic (41% in S-0 and S-1; 36% in M-0 and M-1), binding (30 and 29% in S-0 and M-0, respectively; 31% in S-1 and M-1), structural (6 and 10% in S-0 and M-0, respectively; 5 and 9% in S-1 and M-1, respectively) and transporter activities (3 and 5% in S-0 and M-0, respectively; 4 and 6% in S-1 and M-1, respectively). The remaining categories were significantly less represented, except for proteins with unknown function, which contained 15–17% of the proteins identified. Figure 3 also shows that the distribution of the proteins in functional categories was very similar for every fraction, the only difference being a higher ratio of structural proteins in the insoluble fractions M-0 and M-1. This is mainly due to the higher number of ribosomal proteins in the 100,000× *g* pellets and parallels the distribution observed in the insoluble fractions of the midgut proteome of *O. erraticus* [28].

The biological processes assigned to the identified proteins were similarly represented in the four samples (Fig. 4). The most abundant categories were metabolic processes (31–33%) and single-organism processes (22–25%), followed by biological regulation (7–9%), localization (6–9%), cellular component organization or biogenesis (5–7%), response to stimulus (4–5%) and signal transduction (3%). Finally, proteins with unknown biological process represented 14–15%.

### Comparative analysis of the midgut proteome between unfed and fed *O. moubata* ticks

**Fig. 5:**
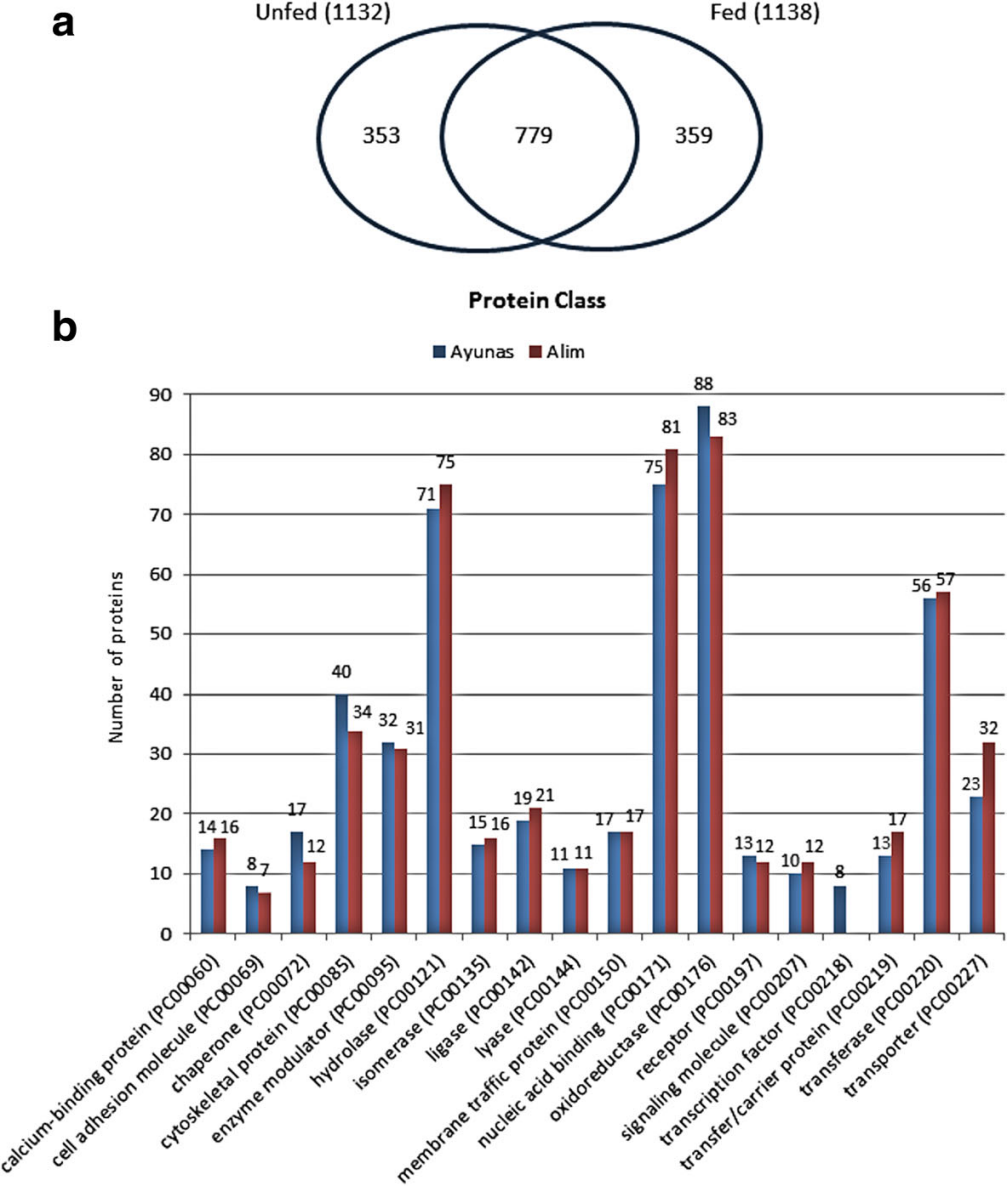
**a** Some proteins identified in the midgut of unfed and fed *Ornithodoros moubata* females. **b** Classification of proteins using the PANTHER system. Only categories with more than five proteins have been included

All proteins identified in the two samples (soluble and membranes) from each physiological condition (fed or unfed) were combined and compared between both physiological conditions (Additional file 2: Table S2). Overall, we identified 1491 non-redundant proteins: 1132 of them in the midgut of unfed ticks, 1138 in the midgut of fed ticks, and up to 779 shared by both physiological conditions (Fig. 5a, Additional file 2: Table S2).

To compare the midgut proteomes before and after feeding, we started by classifying the intestinal proteins expressed in each physiological condition into “protein classes” according to the PANTHER system. This system allowed the classification of 543 and 554 intestinal proteins expressed by the unfed and fed ticks, respectively. Figure 5 shows protein classes containing more than five proteins. The most numerous protein classes were oxidoreductases (88 unfed, 83 fed), nucleic acid binding proteins (75 unfed, 81 fed), hydrolases (71 unfed, 75 fed), transferases (56 unfed, 57 fed), cytoskeletal proteins (40 unfed, 34 fed), enzyme modulator (32 unfed, 31 fed) and transporter proteins (23 unfed, 31 fed). Taken together, these paralleled distributions indicate that the activities and functions of the proteins expressed in the *O. moubata* midgut before and after feeding are very similar, in accordance with what was also observed for the *O. erraticus* midgut [28].

**Fig. 6:**
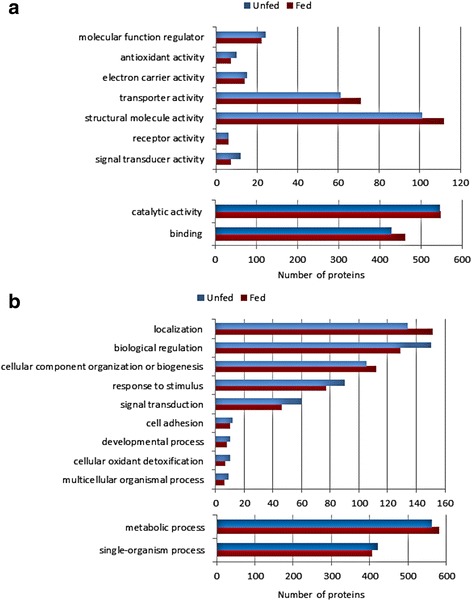
Classification of proteins identified in the midgut of *O. moubata* females before feeding (*blue bars*, unfed) and at 48 h post-feeding (*red bars*, fed). Molecular function (**a**) and biological process (**b**) assigned in the Gene Ontology database

After this analysis, the proteins identified in each physiological condition as having a functional annotation in the Gene Ontology database were classified by their molecular function and biological process. Figure 6 shows that both midgut proteomes, before and after feeding, presented similar patterns. The molecular functions most represented in both conditions were catalytic (63–64%) and binding (50–53%) activities, followed by structural (12–13%) and transporter activities (7–8%), with the remaining categories being minority ones (Fig. 6a). Regarding the biological process, the most numerous were metabolic (69–71%) and single-organism process (50–52%), followed by process of localization (17–19%), biological regulation (16–18%), cellular component organization or biogenesis (13–14%), response to stimulus (10–11%) and signal transduction (6–7%). There were four additional minority categories, each representing around 2% of the proteins (Fig. 6b).

Finally, the active biological pathways in the *O. moubata* midgut were investigated. Thus, the proteins identified in the midgut were further analysed using the KEGG database. We found that proteins expressed in the midgut from unfed and fed female *O. moubata* ticks were involved, respectively, in 108 and 111 enzymatic pathways that clustered into 16 different “Classes” (Table 2, Additional file 3: Table S3).

**Table 2:** Classes of enzymatic pathways and number of proteins in each class identified in the midgut of unfed and fed *Ornithodoros moubata* females. Additional information is shown in Additional file 3: Table S3

Class of enzymatic pathway	Number of proteins	
	Unfed	Fed
Carbohydrate metabolism	193	166
Amino acid metabolism	131	126
Lipid metabolism	124	111
Metabolism of cofactors and vitamins	113	49
Nucleotide metabolism	110	56
Xenobiotics biodegradation and metabolism	106	86
Biosynthesis of antibiotics	87	77
Energy metabolism	81	77
Metabolism of other amino acids	44	38
Immune system	38	21
Biosynthesis of other secondary metabolites	27	19
Metabolism of terpenoids and polyketides	27	24
Glycan biosynthesis and metabolism	22	28
Translation	8	9
Signal transduction	5	4

All these proteins -except the enzymes involved in immune response, translation and signal transduction- participate in the different metabolic pathways that occur in the tick midgut related with the processes of blood digestion and nutrient acquisition [38]. The number of sequences involved in the different pathways indicated that the metabolism of carbohydrates (193 unfed, 166 fed), amino acids (131 unfed, 126 fed) and lipids (124 unfed, 111 fed) were the processes with the highest activity. These data suggest that together with haemoglobin, which is considered the main nutrient source from blood digestion in ticks [38], carbohydrates and lipids would also be important nutrient sources for ticks. This notion is also supported by the *O. moubata* midgut transcriptomic data [29].

The fourth most active enzymatic pathway was the metabolism of cofactors and vitamins, mainly thiamine metabolism, involving 113 enzymes in unfed females and 49 enzymes in fed females (Table 2, Additional file 3: Table S3). The high activity of this pathway in the tick midgut can be somewhat unexpected as these components, cofactors and vitamins, are scarce in the blood of mammals. However, these metabolites can be supplied by the tick intestinal microbiota as has already been demonstrated to occur in the midgut of several haematophagous insects (flies, lice, bugs) and the tick *Amblyomma americanum* [39, 40].

Another notably active enzymatic pathway is nucleotide metabolism, particularly purine metabolism (110 sequences in unfed and 56 in fed ticks) (Table 2, Additional file 3: Table S3). Purine metabolism is very important to tick physiology as it plays a key role in the disposal of nitrogenized waste in the form of guanine [25]. In addition, newly synthesized purines may participate in other functions, such as ribonucleotide synthesis for transcription in the digestive cells, and, furthermore, particular enzymes might act as modulators of the extracellular levels of ATP and ADP to avoid platelet activation and aggregation, and maintain the fluidity of the ingested blood [25, 41].

Overall, the comparative analysis of the midgut proteomes of *O. moubata* females before and after feeding did not reveal great differences in the number or class of proteins expressed, enzymatic composition or functional classification. These data are in accordance with what was also observed in the midgut proteomes of *O. erraticus* and of several ixodid species, which indicates that, at least during the first phases of blood digestion, the proteome remains quite stable and does not experience significant modifications [23, 28, 35]. Conversely, the analysis of tick midgut transcriptomes, including that of *O. moubata*, shows that the expression levels of numerous genes involved in blood digestion and the associated stress responses varied significantly in response to tick feeding [29].

Indeed, it has repeatedly been observed that the dynamics of the transcriptome and proteome of tick midguts are different. Several causes may account for these differential dynamics, including the different sensitivity of the analytical methods (i.e. mass spectrometry *vs* RNA-seq), the different half-life of mRNA and proteins, and the post-transcriptional and post-translational regulation levels of gene expression [36, 42].

### Proteins involved in the blood ingestion and digestion process

Once the midgut proteomes from unfed and fed *O. moubata* females had been characterized and compared, we carried out a more detailed analysis of particular groups of proteins that are involved in the processes of nutrient digestion and responses to the oxidative stress associated with blood ingestion and digestion in ticks. Thus, in accordance with the biological processes in the Gene Ontology database, we selected proteins involved in the following processes: metabolism of proteins, carbohydrates and lipids; endocytoses and intracellular transport; and stress responses and detoxification. The tick midgut is the organ where blood digestion and blood nutrient absorption take place, and this requires that the midgut expresses a range of proteins to perform these tasks [43].

#### Proteolysis

Haemoglobin and serum albumin constitute up to 80% of the blood protein content, and they are the main source of amino acids for ticks, mostly haemoglobin [44]. In ixodids, the process of digesting haemoglobin has been the subject of numerous studies, and it is quite well known [5]. Haemoglobin digestion takes place inside lysosomes or digestive vesicles and is performed by a set of proteolytic enzymes that act sequentially and belong to the following protease families: cysteine peptidases (cathepsin B, L and C, legumain); aspartic endopeptidases (cathepsin D), metallopeptidases (leucine aminopeptidase) and serine peptidases [38]. In *Ixodes ricinus*, the haemoglobinolysis is started by cathepsins D and L and legumain, which cut haemoglobin into large fragments. These fragments are digested by cathepsin B, and secondarily by cathepsin L, into smaller fragments, which finally are degraded into dipeptides and amino acids by cathepsins C and B, a leucine aminopeptidase and a serine carboxypeptidase [15, 45]. Free amino acids reach the haemolymph via transcitosis and are distributed and incorporated into tick tissues [38]. Regarding albumin, it has been recently demonstrated that it is digested by the same proteolytic machinery as haemoglobin [44]. The other blood proteins seem to remain undigested in the gut lumen [38].

In argasids, the information about the molecular machinery of blood digestion is scanter and is limited to the identification of several proteases, including cathepsins B and C, in gut extracts of *O. moubata* [27]. This information has recently been enlarged by Oleaga et al. [28], who identified a cysteine peptidase (legumain), several aspartic endopeptidases, metallopeptidases and serine peptidases in the midgut proteome of *O. erraticus*, all of them likely involved in haemoglobin and albumin digestion.

In the current midgut proteome of *O. moubata,* we identified up to 32 proteases: 5 in unfed females, 9 in fed females and 18 in both conditions. Up to 28 of these proteins were also found in the midgut transcriptome (Table 3). These proteases belong to the same classes/families as described above for ixodids. Thus we have found homologues to cathepsin B, L and D, longipain (which is the cathepsin B of *Haemaphysalis longicornis*), legumain, leucine aminopeptidase and ixodid carboxypeptidase (Table 3) [15, 45]. These results suggest that argasids and ixodids use a very similar proteolytic system to digest host haemoglobin.

**Table 3:** Proteins involved in proteolytic processes identified in the midgut of *Ornithodoros moubata* females before feeding (unfed) and at 48 h post-feeding (fed). (T) Gene identified in the intestinal transcriptome of *O. moubata* [29]

Experimental group	Database	UniProt ID	Protein	emPAI Unfed	emPAI Fed	Molecular function
Unfed	EST_Acari	I3VR81	Aminopeptidase N-10	0.31	–	Metallopeptidase activity
Unfed	EST_Acari	B7PNL8	Cysteine proteinase (T)	2.34	–	Cysteine-type peptidase activity
Unfed	mRNA_Om	B7P458	Glutamate carboxypeptidase (T)	0.04	–	Carboxypeptidase activity
Unfed	NCBI_metazoa	G0WRZ9	Papilin (T)	0.02	–	Metalloendopeptidase activity
Unfed	mRNA_Om	B7PKC5	Sentrin/sumo-specific protease (T)	0.05	–	Endopeptidase activity
Fed	EST_Acari	A0A067R7D1	ATP-dependent metalloprotease YME1L1	–	0.39	Metalloendopeptidase activity
Fed	EST_Acari	E5SL05	Cathepsin B (T)	–	0.47	Cysteine-type peptidase activity
Fed	EST_Acari	R4JNG8	Cathepsin L (T)	–	0.31	Cysteine-type peptidase activity
Fed	mRNA_Om	Q5DNW1	Furin-like convertase (T)	–	0.04	Serine-type endopeptidase activity
Fed	mRNA_Om	B4M1S6	GJ18794 (T)	–	0.07	Serine-type endopeptidase activity
Fed	EST_Acari	XP_010742692	Legumain isoform X2 (T)	–	0.22	Cysteine-type endopeptidase activity
Fed	mRNA_Om	B7P1K5	Prenyl protease (T)	–	0.09	Metalloendopeptidase activity
Fed	mRNA_Om	E9FS53	Putative uncharacterized protein (T)	–	0.04	Serine-type endopeptidase activity
Fed	mRNA_Om	Q6L7Z5	Serine protease (T)	–	0.07	Serine-type endopeptidase activity
Unfed-Fed	EST_Acari	B7PJ08	Aspartic protease (T)	0.59	0.52	Aspartic-type endopeptidase activity
Unfed-Fed	EST_Acari	A0A087TC24	ATP-dependent zinc metalloprotease YME1-like protein	0.24	0.27	Metalloendopeptidase activity
Unfed-Fed	mRNA_Om	G3MKP9	Carboxypeptidase (T)	0.43	0.43	Serine-type carboxypeptidase activity
Unfed-Fed	mRNA_Om	A0A087UZQ9	Caspase-1 (T)	0.25	0.2	Cysteine-type endopeptidase activity
Unfed-Fed	EST_Acari	E7E820	Cathepsin D2	0.59	0.35	Aspartic-type endopeptidase activity
Unfed-Fed	EST_Acari	C6L6E2	Cysteine protease (T)	2.25	1.94	Cysteine-type peptidase activity
Unfed-Fed	mRNA_Om	B4Q2G2	GE16138 (T)	1.47	0.33	Cysteine-type endopeptidase activity
Unfed-Fed	EST_Acari	Q6PRC7	Legumain-like protease (T)	0.33	2.15	Cysteine-type peptidase activity
Unfed-Fed	mRNA_Om	B7QLQ7	Leucine aminopeptidase (T)	0.13	0.2	Metalloexopeptidase activity
Unfed-Fed	EST_Acari	B7PF28	Longipain (T)	0.62	0.44	Cysteine-type endopeptidase activity
Unfed-Fed	EST_Acari	F0J8F6	Metallopeptidase (T)	0.41	0.44	Metallopeptidase activity
Unfed-Fed	mRNA_Om	B7PDF5	Prolyl endopeptidase (T)	0.45	0.56	Serine-type endopeptidase activity
Unfed-Fed	EST_Acari	A0A087UKQ1	Retinoid-inducible serine carboxypeptidase (T)	1.47	1.96	Serine-type carboxypeptidase activity
Unfed-Fed	mRNA_Om	E2AUP0	Serine protease HTRA2, mitocondrial (T)	0.26	0.77	Serine-type endopeptidase activity
Unfed-Fed	mRNA_Om	Q6U8A8	Serine protease-like protein (T)	2.87	2.87	Serine-type endopeptidase activity
Unfed-Fed	mRNA_Om	B7PL27	Signal peptide peptidase (T)	0.25	0.47	Aspartic-type endopeptidase activity
Unfed-Fed	mRNA_Om	B7PQH8	Tripeptidyl-peptidase II (T)	0.22	0.07	Serine-type endopeptidase activity
Unfed-Fed	mRNA_Om	E2BH15	Xaa-Pro aminopeptidase (T)1	1.23	0.16	Metallopeptidase activity

It is worth mentioning that besides their potential digestive functions, some of the proteases identified in the *O. moubata* midgut might have additional functions in other biological processes of the tick. For instance, it has been demonstrated that cathepsin D, found in several ixodid species, participates in defence responses by generating antimicrobial peptides [38, 46]. In the current work, we also found a serine protease in the *O. moubata* midgut (Q6U8A8, Table 3), which contains a sequence motif rich in histidine and aspartic residues similar to that shown by hebraein, an antimicrobial protein from the ixodid tick *Amblyomma hebraeum* [47]. Further studies are needed to confirm whether these *O. moubata* proteins have any function in tick antimicrobial defensive responses.

#### Carbohydrate metabolism and transport

As already indicated by Oleaga et al. [29], information regarding the digestion of carbohydrates and their likely significance as nutrients from blood digestion in ticks is negligible. To date, it has been limited to the identification of several enzymes in the intestinal transcriptomes of four species of ixodids that, owing to their molecular activity, could participate in carbohydrate metabolism [19, 24, 25, 43, 48]. Recently, Oleaga et al. [29] identified up to 113 transcripts in the *O. moubata* midgut transcriptome coding for enzymes involved in carbohydrate metabolism and transport. In the current proteome, we have identified 24 proteins with several enzymatic activities (hydrolase, isomerase, transferase, phosphatase, oxidoreductase and kinase) and 3 proteins with carbohydrate transporter functions (Table 4). Four of them were found in unfed females only, 4 in fed females and 19 were shared by both physiological conditions. Most of these proteins had also been annotated in the transcriptome (Table 4). Two of these proteins show high emPAI values in unfed females. They are two enzymes that participate in the energy metabolism of glucose: the phosphoenolpyruvate carboxykinase (PEPCK) (emPAI 8.58), which is involved in the transformation of glucose in pyruvate in the cell cytoplasm; and malate dehydrogenase (emPAI 19.85), which replaces oxaloacetate in the Krebs cycle. Another important protein in using glucose as an energy source is the glucose transporter 1 (E2C2M2), also identified in the transcriptome, which would be responsible for transporting blood glucose from the intestinal lumen to the inside of the enterocytes (Table 4).

**Table 4:** Proteins involved in carbohydrate metabolism and transport identified in the midgut of *Ornithodoros moubata* females before feeding (unfed) and at 48 h post-feeding (fed). (T) Gene identified in the intestinal transcriptome of *O. moubata* [29]

Experimental group	Database	UniProt ID	Protein	emPAI Unfed	emPAI Fed	Molecular function
Unfed	EST_Acari	B7PDB8	1,4-alpha-glucan branching enzyme	0.62	–	Transferase activity
Unfed	mRNA_Om	B7PDZ5	Alpha-D-galactosidase (T)	0.13	–	Hydrolase activity
Unfed	EST_Acari	A0A0B1PR88	Glucose-6-phosphate isomerase (T)	0.35	–	Isomerase activity
Unfed	mRNA_Om	E2C2M2	Glucose transporter type 1 (T)	0.08	–	Carbohydrate transporter activity
Fed	mRNA_Om	A0A087UZ87	ADP-dependent glucokinase (T)	–	0.05	Transferase activity
Fed	mRNA_Om	Q7Q4V0	AGAP000862-PA (T)	–	0.24	Hydrolase activity
Fed	mRNA_Om	B7PE53	Beta-hexosaminidase (T)	–	0.1	Hydrolase activity
Fed	mRNA_Om	G3MLS5	Putative uncharacterized protein (T)	–	0.65	Hydrolase activity
Unfed-Fed	EST_Acari	B7P5Y3	Alpha-1,4 glucan phosphorylase (T)	1.74	0.1	Transferase activity
Unfed-Fed	EST_Acari	B7QBK7	Alpha-L-fucosidase (T)	0.23	0.33	Hydrolase activity
Unfed-Fed	mRNA_Om	A0A067R124	Alpha-mannosidase (T)	1.25	0.14	Hydrolase activity
Unfed-Fed	mRNA_Om	B7PW84	Beta-galactosidase (T)	5.58	5.51	Hydrolase activity
Unfed-Fed	mRNA_Om	A0A087TZD0	Beta-hexosaminidase subunit alpha (T)	0.85	0.69	Hydrolase activity
Unfed-Fed	mRNA_Om	B7PL94	Beta-mannosidase (T)	0.13	0.09	Hydrolase activity
Unfed-Fed	EST_Acari	B7PLL4	Fructose-1,6-bisphosphatase	1.95	1.22	Hydrolase activity
Unfed-Fed	EST_Acari	E7F0E2	Glucosamine-6-phosphate isomerase (T)	0.98	0.98	Isomerase activity
Unfed-Fed	mRNA_Om	B7PK21	Glucosylceramidase (T)	1.01	0.62	Hydrolase activity
Unfed-Fed	mRNA_Om	A2V728	Glutamine: fructose-6-phosphate aminotransferase (T)	0.06	0.22	Transferase activity
Unfed-Fed	EST_Acari	B7PLJ6	Glycerol-3-phosphate dehydrogenase [NAD(+)] (T)	0.57	0.57	Oxidoreductase activity
Unfed-Fed	mRNA_Om	B7PH44	Malate dehydrogenase (T)	19.85	0.8	Oxidoreductase activity
Unfed-Fed	mRNA_Om	B7QDE7	Mannosyl-oligosaccharide glucosidase (T)	0.27	0.23	Hydrolase activity
Unfed-Fed	mRNA_Om	A9P774	Phosphoenolpyruvate carboxykinase (T)	8.58	0.33	Kinase activity
Unfed-Fed	EST_Acari	A0A087UXS8	Phosphoenolpyruvate carboxykinase [GTP], mitochondrial	3.76	2.95	Kinase activity
Unfed-Fed	mRNA_Om	A0A087TE96	Poly(ADP-ribose) glycohydrolase ARH3(T)	0.65	0.15	Hydrolase activity
Unfed-Fed	mRNA_Om	G3MK74	Putative uncharacterized protein (T)	0.18	0.15	Hydrolase activity
Unfed-Fed	mRNA_Om	B7Q5F0	Transporter (T)	0.08	0.08	Carbohydrate transporter activity
Unfed-Fed	mRNA_Om	E9J2L5	Putative uncharacterized protein (T)	0.11	0.09	Carbohydrate transporter activity

The detection of these enzymes and transporters in *O. moubata*, which are involved in obtaining energy from glucose have recently been described in detail in the intestine of *Ixodes scapularis* [49], suggesting that host blood glucose would also be an important nutrient for both ixodids and argasids.

#### Lipid metabolism and transport

Up to 117 transcripts likely involved in lipid metabolism and transport have been annotated in the midgut transcriptome of *O. moubata* females [29]. Here, we have identified 19 proteins: 3 in unfed females only, 4 in fed females only and 12 in both unfed and fed ticks (Table 5). These consisted of enzymes with various activities (lipase, hydrolase, ceramidase, isomerase, ligase and oxidoreductase) and potential transporters of cholesterol and other lipids.

**Table 5:** Proteins involved in lipid metabolism and transport identified in the midgut of *Ornithodoros moubata* females before feeding (unfed) and at 48 h post-feeding (fed). (T) Gene identified in the intestinal transcriptome of *O. moubata* [29]

Experimental group	Database	UniProt ID	Protein	emPAI Unfed	emPAI Fed	Molecular function
Unfed	mRNA_Om	A0A087U096	Group XV phospholipase A2 (T)	0.14	–	Phospholipase activity
Unfed	mRNA_Om	B7PP53	Triacylglycerol lipase (T)	0.05	–	Lipase activity
Unfed	EST_Acari	B7PV61	Erlin-2	0.31	–	Cholesterol binding
Fed	mRNA_Om	B7Q350	Niemann-Pick type C1 domain-containing protein (T)	–	0.07	Cholesterol transporter activity
Fed	mRNA_Om	Q6QVL7	NPC1 (T)	–	0.33	Cholesterol transporter activity
Fed	mRNA_Om	B7Q6Z1	Saposin (T)	–	0.08	Enzyme activator activity
Fed	mRNA_Om	G9M4L6	Vitellogenin-B (T)	–	0.57	Lipid transporter activity
Unfed-Fed	EST_Acari	E2C1X2	Ankyrin-2 (T)	1.82	0.43	Lipid binding
Unfed-Fed	mRNA_Om	B7QMW0	Fatty acid-binding protein FABP (T)	8.66	0.49	Lipid transporter activity
Unfed-Fed	mRNA_Om	B7PK21	Glucosylceramidase (T)	1.01	0.62	Glucosylceramidase activity
Unfed-Fed	mRNA_Om	B7PG19	Inositol-1-phosphate synthetase (T)	0.07	0.04	Isomerase activity
Unfed-Fed	mRNA_Om	B7PJA8	Long chain fatty acid CoA ligase (T)	1.61	1.55	Ligase activity
Unfed-Fed	mRNA_Om	B7QDC5	Low-density lipoprotein receptor, putative (T)	3.71	0.17	Hydrolase activity
Unfed-Fed	mRNA_Om	B7P1A9	Major epididymal secretory protein HE1 (T)	3.43	0.86	Cholesterol transporter activity
Unfed-Fed	mRNA_Om	B7Q8J7	N-acylsphingosine amidohydrolase (Acid ceramidase) (T)	1.43	0.71	Ceramidase activity
Unfed-Fed	mRNA_Om	B7Q381	Putative uncharacterized protein (T)	0.24	0.24	Transporter activity
Unfed-Fed	mRNA_Om	B7PYU9	Rat sphingolipid delta 4 desaturase (T)	0.06	0.09	Oxidoreductase activity
Unfed-Fed	mRNA_Om	B6ZIV7	Vitellogenin (T)	1.65	0.57	Lipid transporter activity
Unfed-Fed	mRNA_Om	E1CAX9	Vitellogenin-1 (T)	13.21	0.33	Lipid transporter activity

Among the enzymes, there were group XV phospholipase A2, triacylglycerol lipase, inositol-1-phosphate synthetase, acylsphingosine amidohydrolase and sphingolipid desaturase (Table 5). Group XV phospholipase A2 (A0A087U096) is different from the secreted phospholipase A2 identified in *O. moubata* saliva [50] and from the phospholipase A2 identified in the midgut transcriptome of *O. moubata* [29]. This group XV phospholipase A2 is known as lysosomal phospholipase because it is found inside lysosomes and it participates in the degradation of endogenous phospholipids [51].

Among lipid transporters, we found four proteins whose function was cholesterol binding and transport (Table 5). Unlike vertebrates, arthropods cannot synthesize cholesterol themselves and must acquire it from their diet, as it is necessary for the biosynthesis of the ecdysteroids or hormones regulating the processes of development and reproduction [25, 52]. One of these proteins is the low-density lipoprotein (LDL) receptor (B7QDC5). LDL is the main plasma cholesterol-carrying lipoproteins. This receptor, which is expressed on the membrane of the intestinal cell, captures LDL molecules and transports them into the enterocytes by a mechanism of endocytosis involving clathrin-coated vesicles. Once inside the cell, free cholesterol binds to endogenous tick proteins, passes to the haemolymph and is distributed throughout the remaining tick tissues [25]. Up to seven transcripts coding for this LDL receptor were identified in the *O. moubata* midgut transcriptome, lending additional support to the importance of this mechanism in the *O. moubata* physiology.

Three additional proteins likely engaged in the transport of cholesterol are the Niemann-Pick type C1 (NPC1) proteins (B7Q350, Q6VL7) and the epididymal secretory protein E1 (also known as Niemann-Pick C2 protein - NPC2) (Table 5) [53]. Likewise, erlin-2 is a protein that localizes to areas of lipid accumulation of the endoplasmic reticulum membrane and regulates cholesterol levels inside the cell (Table 5) [54, 55].

Other proteins showing lipid transport function are ankyrin-2 (E2C1X2), three vitellogenins (G9M4I6, B6ZIV7, E1CAX9), a fatty acid transport protein (B7QMW0) and an uncharacterized protein (B7Q381). Ankyrin is a protein that has a domain common to the family of apolipoproteins, which, together with the vitellogenins, belongs to a large protein family of lipid transporters. Vitellogenin, also involved in binding and detoxification of the haem group, is the precursor of vitellin, which is the main component of eggs and provides the necessary energy and amino acids for egg development and oviposition [56, 57] (Table 5).

#### Endocytosis and intracellular transport

The haemoglobin released after erythrocyte lysis is incorporated into the digestive cells through an endocytic process, mediated by specific receptors present in clathrin-coated vesicles that are internalized to form large endosomes. Albumin is also internalized by endocytosis, but using a non-specific transport mechanism that does not require the involvement of clathrin. The proteolytic enzymes that digest haemoglobin, and likely the albumin, are synthesized as precursors in the endoplasmic reticulum and are transferred to lysosomal vesicles through the Golgi. The lysosomes and the endosomes that contain haemoglobin fuse to form the digestive vesicles where proteolysis takes place [5, 15, 43].

Up to 43 proteins likely involved in the recognition and internalization of haemoglobin inside endosomes, the trafficking and fusion of intracellular vesicles, and the transport of proteins and other molecules inside the digestive cells were identified in the *O. moubata* midgut proteome: 8 of these proteins were found in unfed females, 14 in fed females and 21 in both physiological conditions (Table 6).

**Table 6:** Proteins involved in endocytosis and intracellular transport identified in the midgut of *Ornithodoros moubata* females before feeding (unfed) and at 48 h post-feeding (fed). (T) Gene identified in the intestinal transcriptome of *O. moubata* [29]

Experimental group	Database	UniProt ID	Protein	emPAI Unfed	emPAI Fed	Biological process
Unfed	EST_Acari	G9F9U2	ABC C2 transporter	0.26	–	Transmembranne transport
Unfed	EST_Acari	B7PAI1	Acetylcholine regulator unc-18	1.47	–	Vesicular transport
Unfed	EST_Acari	A0A087U0D4	AP-1 complex subunit beta-1 (T)	0.7	–	Vesicular transport
Unfed	EST_Acari	A0A087TIH9	AP-1 complex subunit sigma-2 (T)	0.44	–	Vesicular transport
Unfed	EST_Acari	A0A087UKS9	AP-2 complex subunit alpha-2 (T)	0.26	–	Vesicular transport
Unfed	NCBI_metazoa	A0A087TIC9	Clathrin heavy chain 1 (T)	0.03	–	Vesicular transport
Unfed	EST_Acari	F0J8C0	Glucose derepression and pre-vacuolar endosome protein sorting protein	0.3	–	Vacuolar transport
Unfed	mRNA_Om	B7PRM4	VAMP-7 (T)	0.22	–	Endocytosis
Fed	mRNA_Om	Q7QHU5	AGAP011358-PA	–	0.26	Vesicular transport
Fed	mRNA_Om	B7PEY0	AP-2 complex subunit alpha-1 (T)	–	0.38	Vesicular transport
Fed	EST_Acari	Q6WCQ8	Beta-adaptin (T)	–	0.37	Vesicular transport
Fed	EST_Acari	V9TLV5	Clathrin (T)	–	0.37	Vesicular transport
Fed	mRNA_Om	Q8T9S5	Clathrin-adaptor protein (T)	–	0.2	Vesicular transport
Fed	NCBI_metazoa	A0A090L934	Coatomer subunit alpha (T)	–	0.23	Vesicular transport
Fed	EST_Acari	B7PXJ9	COPII vesicle protein	–	0.27	Vesicular transport
Fed	mRNA_Om	Q6QT18	KOG1656-like protein (T)	–	0.17	Vacuolar transport
Fed	EST_Acari	B7PMB5	Phosphatidylinositol-binding clathrin assembly protein (T)	–	0.23	Clathrin coat assembly
Fed	EST_acari	D6WJQ8	Rab-protein 5	–	0.76	Vesicular transport
Fed	mRNA_Om	B7PES3	Vesicle coat complex AP-3, delta subunit (T)	–	0.14	Vesicular transport
Fed	EST_Acari	F0J8Z5	Vesicle coat complex COPI beta’ subunit (T)	–	0.25	Vesicular transport
Fed	mRNA_Om	A0A087TZJ7	Vesicle transport through interaction with t-SNAREs-like protein 1B (T)	–	0.12	Vesicular transport
Fed	mRNA_Om	A0A087TTT4	Vesicle-associated membrane protein 1 (T)	–	0.09	Vesicular transport
Unfed-Fed	mRNA_Om	Q3YB24	ABC transporter ABCA1 (T)	0.07	0.04	Transmembranne transport
Unfed-Fed	mRNA_Om	B7PUI6	ABC transporter (T)	6.52	3.87	Transmembranne transport
Unfed-Fed	mRNA_Om	B7Q6V9	AP complex subunit beta (T)	0.48	0.45	Vesicular transport
Unfed-Fed	mRNA_Om	B7P454	Cargo transport protein EMP24 (T)	0.62	0.92	Protein transport
Unfed-Fed	mRNA_Om	B7PUK8	Clathrin heavy chain (T)	0.57	0.77	Vesicular transport
Unfed-Fed	mRNA_Om	A0A087U9S2	Coatomer subunit beta (T)	0.37	0.84	Vesicular transport
Unfed-Fed	mRNA_Om	A0A087UHC8	Coatomer subunit gamma-2 (T)	0.38	0.61	Vesicular transport
Unfed-Fed	mRNA_Om	B7PM12	Dynamin (T)	0.13	0.1	Clathrin-dependent endocytosis
Unfed-Fed	EST_Acari	B7P6P0	Glycoprotein 25 l	0.9	0.91	Protein transport
Unfed-Fed	EST_Acari	B7QNW0	Protein required for fusion of vesicles in vesicular transport, alpha-SNAP	0.46	0.49	Intracellular protein transport
Unfed-Fed	EST_Acari	Q6XP57	Rab11–2	1.87	1.4	Vesicular transport
Unfed-Fed	EST_Acari	B7QFX7	RAB-9 and, putative	3.64	3.67	Vesicular transport
Unfed-Fed	mRNA_Om	A0A087TXJ3	Rab GDP dissociation inhibitor beta (T)	2.33	1.51	Protein transport
Unfed-Fed	mRNA_Om	B7PAE0	RAS-related protein (T)	0.16	0.29	Regulation of endocytosis
Unfed-Fed	mRNA_Om	B7Q8P2	Sorting nexin (T)	0.15	0.44	Endocytosis
Unfed-Fed	mRNA_Om	B7PZR4	Surfeit 4 protein (T)	0.22	0.63	Vesicular transport
Unfed-Fed	EST_Acari	A0A023JCU7	Synaptobrevin (P)	0.18	0.28	Vesicular transport
Unfed-Fed	mRNA_Om	B7P427	Transmembrane protein Tmp21 (T)	2.0	1.74	Protein transport
Unfed-Fed	EST_Acari	B7P164	Vacuolar protein sorting-associated protein 29 (T)	0.52	0.46	Intracellular protein transport
Unfed-Fed	mRNA_Om	B7QLI1	Vacuolar sorting protein (T)	0.09	0.17	Intracellular protein transport
Unfed-Fed	EST_Acari	B7PDY5	Vesicle coat complex COPII, GTPase subunit SAR1 (T)	0.81	1.65	Vesicular transport

Among the proteins potentially involved in the endocytosis process, and the formation and assembly of clathrin-coated vesicles, we identified several clathrins: three proteins belonging to the adaptor protein (AP) complex (AP-1, AP-2 and AP-3) and a beta-adaptin [58]. In relation to the regulation of these mechanisms, we also identified the phosphatidylinositol-binding clathrin assembly protein, which limits the size of clathrin-coated vesicles and controls the traffic of endocytic membranes by recruiting adapters and other components of the transport machinery [59]. Dynamin-1 plays a key role in the fusion of the endocytic membrane [60] (Table 6).

Among the proteins that may be implicated in vesicle-mediated intracellular transport, we identified, among others, the following: acetylcholine regulator unc-18; alpha-SNAP; synaptobrevin and its homologue VAMP-7; the Rab family of proteins; transport-interaction with t-SNAREs-like protein 1B; surfeit 4; the coatomers COPI and COPII; cargo transport protein EMP24; transmembrane protein Tmp21 and glycoprotein 25 l (Table 6).

The acetylcholine regulator unc-18 protein regulates vesicle transport by preventing the formation of the SNARE complex, which, together with the alpha-SNAP protein, synaptobrevin and surfeit 4, is essential for specific fusion between the membranes of the vesicles [61, 62]. Rab proteins are a family of GTPases that regulate the intracellular transport of vesicles, directing the trafficking and fusion of the vesicle to the target membrane, thus fulfilling a key role in the specificity of the vesicular transport within the cell [63].

Coatomers COPI and COPII (coat complex proteins) are multiprotein complexes that, when assembled, form a coating that covers the vesicles that transport proteins and lipids, and mediates their trafficking between the Golgi apparatus and the endoplasmic reticulum [64, 65] (Table 6). Related to this latter mechanism are the proteins cargo transport protein EMP24, transmembrane protein Tmp21 and glycoprotein 251, which are members of the p24 family. This is a very conserved family of membrane proteins that mediate the assembly of COPI coatomers in the membranes of vesicles [66].

Regarding the intracellular transport of molecules, it has recently been demonstrated that the “ABC transporter” proteins mediate the transport of the haem group from the digestive vesicles to the haemosomes, where it accumulates to form large aggregates [67]. We have identified three of these ABC transporters: G9F9U2, Q3YB2 and B7PUI6. On the other hand, the surfeit 4 protein, besides being involved in the fusion of membranes, is also believed to be involved in the transport of haemoglobin in the cell cytosol [38].

Some of the above-listed proteins were also identified in the intestinal proteomes of *Rhipicephalus microplus* and *O. erraticus*, which revealed the importance of mechanisms of intracellular transport within the enterocytes during blood digestion processes [21, 28]. Our results significantly expand the knowledge of the elements that are part of the intracellular transport machinery, highlighting its complexity and the interest for deepening the knowledge of the regulatory mechanisms that mediate these processes.

#### Oxidative stress response and detoxification

The digestion of haemoglobin inside digestive vesicles of enterocytes releases large amounts of haem group, iron ions, hydrogen peroxide, hydroxyl radicals and other toxic molecules that induce oxidative responses. As other haematophagous organisms, ticks have detoxifying mechanisms that block the oxidative reactions produced by blood digestion, thus protecting themselves from their deleterious effects [68–70]. One of these detoxifying mechanisms developed by ticks is the accumulation of excess haem molecules in intracellular organelles called haemosomes [71].

In the midgut transcriptome of *O. moubata*, Oleaga et al. [29] identified up to 79 genes involved in response to cellular oxidative stress, most of which were upregulated after feeding, in parallel with what was also observed in the mialome of *Dermacentor marginatus* [19].

In the midgut proteomes analysed in the current work, we identified up to 13 antioxidant proteins with oxidoreductase activity, 12 proteins involved in detoxification processes, up to 40 heat shock proteins (HSPs), also known as stress response proteins, and other chaperones involved in protein folding processes (Table 7).

**Table 7:** Proteins involved in the stress responses associated to blood digestion that were identified in the midgut of *Ornithodoros moubata* females before feeding (unfed) and at 48 h post-feeding (fed). (T) Gene identified in the intestinal transcriptome of *O. moubata* [29]

Experimental group	Database	UniProt ID	Protein	emPAI Unfed	emPAI Fed	Molecular function
Antioxidants						
Unfed	EST_Acari	B7Q8W6	Alkyl hydroperoxide reductase, thiol specific antioxidant	0.53	–	Oxidoreductase activity
Unfed	mRNA_Om	B7QGB0	Peroxidase (T)	0.09	–	
Unfed	mRNA_Om	B7PUM7	Peroxinectin (T)	0.1	–	
Unfed	NCBI_metazoa	Q9GV35	Peroxiredoxin (T)	0.46	–	
Unfed	EST_Acari	B7QN17	Thioredoxin-dependent peroxide reductase (T)	0.99	–	
Fed	EST_Acari	T2FDK5	Catalase (T)	–	1.45	
Fed	EST_Acari	A6N9S1	Thioredoxin peroxidase (T)	–	1.84	
Unfed-Fed	EST_Acari	F0JAD0	Dihydrolipoamide dehydrogenase	2.44	0.32	
Unfed-Fed	EST_Acari	Q2XW17	Glutathione peroxidase (T)	1.65	1.1	
Unfed-Fed	EST_Acari	B7PUC4	Superoxide dismutase [Cu-Zn] (T)	4.58	2.15	
Unfed-Fed	mRNA_Om	E2ASR9	Thioredoxin-like protein 1 (T)	1.68	0.17	
Unfed-Fed	EST_Acari	A6NA14	Truncated peroxiredoxin	1.04	0.61	
Unfed-Fed	mRNA_Om	B7PTG8	Thioredoxin reductase (T)	0.53	0.04	
Chaperones						
Unfed	EST_Acari	B7Q6Y2	Chaperonin subunit	0.58	–	Protein folding
Unfed	EST_Acari	A0A097A1J8	Heat shock 70 kDa protein	18.61	–	
Unfed	NCBInr	gi|557,767,195	heat shock 70 kDa protein cognate 4-like	0.2	–	
Unfed	NCBI_metazoa	Q4ZJ79	Heat shock cognate 70 protein (T)	0.2	–	
Unfed	EST_Acari	B7PEU9	Heat shock protein (T)	0.35	–	
Unfed	EST_Acari	B7PIN1	Heat shock protein 20.6 (T)	0.27	–	
Unfed	NCBI_metazoa	Q5I5Q6	Heat shock protein 60 (T)	0.23	–	
Unfed	EST_Acari	B7QI01	Hsp90 protein (T)	2.82	–	
Unfed	mRNA_Om	G3MRN7	Putative uncharacterized protein	0.27	–	
Unfed	EST_Acari	B7P3Z6	T-complex protein 1 subunit gamma (T)	1.06	–	
Unfed	NCBInr	E4WZX2	Whole genome shotgun assembly, reference scaffold set	0.08	–	
Fed	EST_acari	F0 J987	Endoplasmic reticulum glucose-regulated protein	–	1.8	
Fed	mRNA_Om	B4JT04	GH23301	–	0.17	
Fed	EST_acari	E4W3Z2	Heat shock 70 kDa protein 5 (T)	–	8.27	
Fed	NCBInr	B4YTT9	Heat shock protein 70–2 (T)	–	0.19	
Fed	EST_Acari	F0J8P3	HSP70 family member (T)	–	7.42	
Fed	mRNA_Om	B7Q150	Molecular chaperone	–	0.1	
Fed	mRNA_Om	G3MQW7	Putative uncharacterized protein	–	0.21	
Fed	mRNA_Om	B7PN00	TPR domain-containing protein (T)	–	0.11	
Fed	NCBI_metazoa	U4TVI5	Uncharacterized protein	–	0.1	
Unfed-Fed	EST_Acari	B7PGQ2	Calnexin	0.51	0.67	
Unfed-Fed	EST_Acari	Q68HD1	Calreticulin	1.4	0.73	
Unfed-Fed	EST_Acari	B7PZ24	Chaperonin complex component, TCP-1 delta subunit	0.27	0.23	
Unfed-Fed	EST_Acari	B7QJ21	Chaperonin complex component, TCP-1 eta subunit	0.23	1.67	
Unfed-Fed	mRNA_Om	G6DGE9	Chaperonin containing t-complex polypeptide 1 beta subunit	4.89	6.06	
Unfed-Fed	EST_Acari	B7PEV0	Chaperonin subunit	1.11	1.04	
Unfed-Fed	EST_Acari	F0J8S6	FKBP-type peptidyl-prolyl cis-trans isomerase	0.44	0.26	
Unfed-Fed	mRNA_Om	B4KRE5	GI20465	1.34	0.91	
Unfed-Fed	EST_Acari	A0A087TSI6	GrpE protein homolog	0.36	0.85	
Unfed-Fed	mRNA_Om	B7PPJ5	Grpe protein	0.34	0.47	
Unfed-Fed	EST_Acari	B7PWF5	Heat shock protein 20.5 (T)	3.55	0.47	
Unfed-Fed	mRNA_Om	B7Q0B3	Heat shock protein 70 (HSP70)-interacting protein (T)	0.59	0.54	
Unfed-Fed	NCBI_metazoa	M9WB33	Heat shock protein 90	0.21	3.51	
Unfed-Fed	EST_Acari	F0J8Z3	Mitochondrial chaperonin Cpn60/Hsp60p	2.85	1.16	
Unfed-Fed	EST_Acari	B7PD56	Peptidyl-prolyl cis-trans isomerase (T)	4.59	0.66	
Unfed-Fed	mRNA_Om	G3MKV9	Putative uncharacterized protein	1.37	1.42	
Unfed-Fed	EST_Acari	A0A087UTZ9	T-complex protein 1 subunit alpha (T)	0.71	0.97	
Unfed-Fed	mRNA_Om	B7PRH5	T-complex protein 1 subunit delta (T)	2.04	4.61	
Unfed-Fed	EST_Acari	B7QC85	Tumor rejection antigen (Gp96)	1.29	1.32	
Unfed-Fed	mRNA_Om	H9JT91	Uncharacterized protein (T)	2.64	6.15	
Detoxification						
Unfed	mRNA_Om	Q9Y1T8	Cytochrome P450 4 W1 (T)	0.37	–	Heme binding
Unfed	mRNA_Om	B7P9Q4	Cytochrome P450 (T)	0.05	–	
Fed	EST_Acari	B7P5J0	Cytochrome P450 (T)	–	0.74	
Fed	mRNA_Om	B7QES0	Cytochrome P450 (T)	–	1.18	
Fed	mRNA_Om	B7QJS5	Cytochrome P450 (T)	–	0.23	
Unfed-Fed	mRNA_Om	B7PME9	Cytochrome P450 (T)	0.98	1.78	
Unfed-Fed	mRNA_Om	B7PG84	Glutathione S-transferase (T)	21.84	2.09	Transferase activity
Unfed-Fed	EST_Acari	B7PR98	Glutathione S-transferase (T)	4.24	4.97	
Unfed-Fed	mRNA_Om	B7Q917	Glutathione S-transferase (T)	0.8	0.69	
Unfed-Fed	mRNA_Om	B7PRA0	Glutathione S-transferase (T)	2.54	0.33	
Unfed-Fed	mRNA_Om	B7QMI2	Glutathione S-transferase (T)	9.09	0.49	

Related to the antioxidant enzymes responsible for removing hydrogen peroxide, we identified several thioredoxin peroxidases (peroxiredoxins) (Q9GV35, A6N9S1, A6NA14), thioredoxin reductases (B7QN17, B7PTG8), peroxidases (B7Q8W6, B7QGB0), peroxinectin (B7PUM7), glutathione peroxidase (Q2XW17), catalase (T2FDK5) and superoxide dismutase (SOD) (B7PUC4) (Table 7). Some of these proteins have also been identified in the gut of *O. erraticus* and several ixodid species, where they participate in the defence against cellular oxidative stress [19, 21, 25, 28, 43, 70]. Specifically, catalase is the main enzyme responsible for controlling hydrogen peroxide released in digestive cells after blood ingestion [70]. On the other hand, among the enzymes with oxidoreductase activity, the enzyme SOD stands out due to its abundance, especially in the unfed ticks (emPAI 4.59 in unfed and 2.15 in fed ticks). Besides its antioxidant properties of removing free radicals produced during blood digestion, SOD is likely involved in additional biological processes because it can also regulate the size of the populations of the pathogens transmitted by the tick [72] and participate in transporting the haem group inside the digestive cells [19, 73].

HSPs and other chaperones are a set of highly conserved proteins produced by cells whose concentration increases in stressful situations, such as the increase in temperature after blood ingestion at 37 °C. In such a situation of stress exposure, these proteins stabilize other proteins inside cells, preventing their denaturation, and promoting their folding and assembly to generate the correct tertiary structure [74]. Many upregulated transcripts coding for HSPs and chaperones were identified in the midgut transcriptome of *O. moubata* after feeding [29].

Table 7 shows the numerous chaperones identified in the *O. moubata* midgut proteome. Among them, the following proteins are noteworthy because of their abundance: three HSPs of 70 kDa (A0A097A1J8, E4W3Z2, F0J8P3) with emPAI values of 18.61, 8.27 and 7.42; the chaperonin containing t-complex polypeptide (G6DGE9) protein with emPAI values of 4.89 in unfed and 6.06 in fed; and the peptidyl-prolyl cis-after isomerase protein (B7PD56) with an emPAI value in unfed ticks of 4.59. HSP70 not only contributed to cell protection against the stress generated by blood digestion, but it also could participate in removing the clathrin-cover from the endocytic vesicles, thus playing a significant role in the intracellular vesicular transport [75].

Several proteins involved in detoxification processes were also identified in the *O. moubata* midgut proteome, including members of the family of cytochrome P450 (CYPs) and members of the family of glutathione S-transferases (GSTs) (Table 7). CYPs are involved in phase I of the xenobiotic detoxification system. In *O. moubata*, we identified up to five components of this family. These enzymes have a haem group in their structure that acts as a prosthetic group. This might suggest some relation of CYPs to the digestion of haemoglobin; however, it has recently been shown that, at least in *Ixodes ricinus*, their expression is independent of blood ingestion [25].

The GST family comprises several classes of enzymes, some of which participate in phase II of the detoxification system [25]. We have identified up to five GSTs in the midgut of *O. moubata*, which were also present in the *O. erraticus* midgut [28] (Table 7).

## Conclusions

This work, together with the former analysis of the *O. moubata* midgut transcriptome [29], have provided a wealth of unprecedented information on the genes and proteins involved in the digestion process in argasids, particularly in the processes of nutrient metabolism and transport and the defensive, detoxifying and antioxidant responses triggered by the ingestion of blood. The identification of a set of proteolytic enzymes belonging to the same classes/families of proteases in the midgut of the argasid tick *O. moubata* as those described in ixodid ticks, reveals that the haemoglobinolytic system in both tick families is very similar although they display very different feeding and reproductive strategies. Although the main source of nutrients during host blood digestion in ticks is proteins, particularly haemoglobin and albumin, the identification of numerous genes and proteins involved in the metabolism and transport of lipids and carbohydrates reveals that these components also constitute significant nutritional sources and play an important part in the process of blood digestion. The genes and proteins identified in the mialome of *O. moubata* that are involved in intracellular transport mechanisms, defensive responses, detoxifying responses and stress responses seem to be closely regulated, highlighting the complexity and importance of these processes in tick biology, which in turn assigns them great interest as targets for therapeutic and/or immunological interventions. Our results also confirm the usefulness of the transcriptome as a searchable database for improving identifications in proteomic analyses, especially in those carried out on organisms that do not have their genome sequenced, as is the case for *O. moubata*.

## Additional files

Additional file 1: **Table S1.** Non-redundant host proteins identified in Soluble fraction, S-0, from midgut of unfed *Ornithodoros moubata* female ticks (XLSX 26 kb)

Additional file 2: **Table S2.** Non-redundant tick proteins identified in the midgut of unfed ticks (unfed group) and engorged ticks at 48 h post-feeding (fed group) (XLSX 295 kb)

Additional file 3: **Table S3.** Biological pathways represented in the midgut unfed ticks, number of sequences and enzymes involved in each pathway. The analysis of the midgut proteome was done in the KEEG pathway database using the Blast2GO software (XLSX 44 kb)

## Abbreviations

ACN: Acetonitrile
AP: Adaptor protein
COP: Coat complex proteins
CYP: Cytochrome P450
DTT: Dithiothreitol
emPAI: Exponentially modified protein abundance index
EST: Expressed sequence tag
FA: Formic acid
GO: Gene Ontology
GST: Glutathione S-transferases
HSP: Heat shock protein
IDA: Information dependent acquisition
KEGG: Kyoto Encyclopedia of Genes and Genomes
LC/MS-MS: Liquid chromatography tandem mass spectrometry
LDL: Low-density lipoprotein
M-0: Membrane-associated proteins from unfed ticks
M-1: Membrane-associated proteins from fed ticks
mRNA: Messenger ribonucleic acid
NCBInr: National Center for Biotechnology Information non-redundant
PANTHER: Protein Analysis Through Evolutionary Relationships
PBS: Phosphate buffered saline
PIT: Proteomics informed by transcriptome
RNAseq: Ribonucleic acid sequencing
S-0: Soluble proteins from unfed ticks
S-1: Soluble proteins from fed ticks
SDS-PAGE: Sodium dodecyl sulfate polyacrylamide gel electrophoresis
SOD: Superoxide dismutase
TFA: Trifluoroacetic acid
TOF MS: Time of flight mass spectrometry
